# Applications of Machine Learning to Improve the Clinical Viability of Compton Camera Based *in vivo* Range Verification in Proton Radiotherapy

**DOI:** 10.3389/fphy.2022.838273

**Published:** 2022-04-11

**Authors:** Jerimy C. Polf, Carlos A. Barajas, Stephen W. Peterson, Dennis S. Mackin, Sam Beddar, Lei Ren, Matthias K. Gobbert

**Affiliations:** 1 Department of Radiation Oncology, University of Maryland School of Medicine, Baltimore, MD, United States,; 2 Department of Mathematics and Statistics, University of Maryland Baltimore County, Baltimore, MD, United States,; 3 Department of Physics, University of Cape Town, Rondebosch, South Africa,; 4 Department of Medical Physics, University of Texas M.D. Anderson Cancer Center, Houston, TX, United States

**Keywords:** prompt gamma, compton camera, proton radiotherapy, range verification, *in vivo* imaging, proton pencil beam

## Abstract

We studied the application of a deep, fully connected Neural Network (NN) to process prompt gamma (PG) data measured by a Compton camera (CC) during the delivery of clinical proton radiotherapy beams. The network identifies 1) recorded “bad” PG events arising from background noise during the measurement, and 2) the correct ordering of PG interactions in the CC to help improve the fidelity of “good” data used for image reconstruction. PG emission from a tissue-equivalent target during irradiation with a 150 MeV proton beam delivered at clinical dose rates was measured with a prototype CC. Images were reconstructed from both the raw measured data and the measured data that was further processed with a neural network (NN) trained to identify “good” and “bad” PG events and predict the ordering of individual interactions within the good PG events. We determine if NN processing of the CC data could improve the reconstructed PG images to a level in which they could provide clinically useful information about the *in vivo* range and range shifts of the proton beams delivered at full clinical dose rates. Results showed that a deep, fully connected NN improved the achievable contrast to noise ratio (CNR) in our images by more than a factor of 8x. This allowed the path, range, and lateral width of the clinical proton beam within a tissue equivalent target to easily be identified from the PG images, even at the highest dose rates of a 150 MeV proton beam used for clinical treatments. On average, shifts in the beam range as small as 3 mm could be identified. However, when limited by the amount of PG data measured with our prototype CC during the delivery of a single proton pencil beam (~1 × 10^9^ protons), the uncertainty in the reconstructed PG images limited the identification of range shift to ~5 mm. Substantial improvements in CC images were obtained during clinical beam delivery through NN pre-processing of the measured PG data. We believe this shows the potential of NNs to help improve and push CC-based PG imaging toward eventual clinical application for proton RT treatment delivery verification.

## 1 INTRODUCTION

Proton radiotherapy (RT) has shown several advantages in dose conformity, tumor control probability, and normal-tissue complications over conventional RT such as x-ray or electron therapy [1–3]. However, limitations in our ability to accurately determine the position of the proton Bragg peak (BP) during planning, and to verify that it matches the actual BP position and range of the beam in the patient during treatment, have thus far limited the ability of RT practitioners to take full advantage of the high conformality and steep distal dose gradients achievable with proton RT [4–6]. These limitations in our ability to calculate/determine the beam range and BP position can result in an overshoot or undershoot of the tumor. This can lead to under dosage of the tumor or delivery of unsafe doses to healthy organs and tissues adjacent to the tumor. To help detect and avoid such delivery errors, many researchers have studied techniques for range verification of proton treatment beams [7–18].

Compton cameras (CC) have been widely studied as a tool to image secondary prompt gammas (PG) emitted along the proton beam path as one potential method for verifying the range of the proton beam within the patient during proton RT treatment delivery [7]. CCs are multistage detectors that use the principles of Compton scattering [19] to measure the energy deposition and position for each interaction of a gamma as it scatters in the different detection stages of the camera. From the energy deposition and position data for each gamma scatter the gamma’s incident energy and the angle of its initial scatter in the detector can be determined [20–24]. The location of the first two interactions in the CC defines the central axis, and the calculated scatter angle defines the opening angle of the PG “cone-of-origin” with an apex located at the point of the first interaction. The true point of emission for the PG is restricted to lie somewhere on the surface of its cone-of-origin. By backprojecting the cones-of-origin for multiple PGs through the imaging space, an image of the PG emission along the path of the proton beam can be reconstructed.

The use of CCs for proton beam range verification is of particular interest due to their ability to reconstruct full 3D images of PG emission, which could, in principle, be registered and overlaid onto the patients’ CT dataset for visual (and analytical) comparison to the planned treatment dose [8, 11]. While 3D image reconstruction of PG emission with a CC during proton beam delivery has been proven feasible [25, 26], the ability to do so at full clinical proton RT dose rates and under full clinical treatment conditions has thus far not been possible. Several studies of prototype CCs with high energy accelerator beams and clinical proton beams have shown rather poor performance for detecting the “true” double-scatter (DS; a single PG interacting twice in the CC, including Compton—photo-absorption, Compton—Compton, and Compton—pair production interactions) and “true” triple-scatter (TS; a single PG interacting three times in the CC, including two Compton interactions and a third Compton, photo-absorption, or pair production interaction) PG events needed for CC image reconstruction [25, 27–30]. This poor performance is due to: 1) inherently poor efficiency of most prototype CCs for detecting DS and TS events, 2) high detector dead time encountered by many types of CCs caused by the large signal environment encountered during proton RT, 3) interactions of secondary particles other than PGs [31, 32], 4) “mis-ordered” DS and TS events whose individual interactions in the CC are read out and recorded in the wrong order, 5) the detection of “false” events (sometimes referred to as “fortuitous”, “background”, “chance”, or “random” coincidence events), which are DS or TS events that are due to more than one PG interacting simultaneously in the CC [31–34] and 6) “double-to-triple” (D-to-T) events, which occur when a true DS and single-scatter from a separate PG are recorded together as a TS event.

Several studies [31, 35, 36] have shown that mis-ordered, false, and D-to-T events do not contribute to the image signal and act only to increase noise and reduce the achievable contrast of the image. Methods to determine correct event ordering [24, 36, 37] based on classical Compton kinematics have been studied. However, no efficient method has been developed to identify the correct interaction order of DS or TS events in which the initial PG energy is not known (or assumed) a priori. Recent studies have shown how CC imaging can still be improved through improving data acquisition and readout electronics [35], and that machine learning, in particular Neural Networks, can be used to pre-process the PG event data prior to image reconstruction. In particular, Zoglauer et al. [38] and Basalyga et al. [39] showed that relatively simple NNs can be used to predict the correct ordering of TS interactions in a CC. Also, Muñoz et al. [26] showed that simple NNs can be used to identify true and false TS events recorded by a CC during delivery of experimental, low intensity proton beams and that using the NN predicted true TS events led to modest improvements in the final images.

In this paper, we report on the use of a more complex deep, fully connected NN [39, 40] for expanded types of pre-processing of PG data measured with a CC during delivery of a clinical proton RT beam to a tissue equivalent target. This NN was trained to 1) identify true and false DS/TS events, 2) identify the correct interaction ordering of true DS/TS events, and 3) to identify the DS event (and its correct interaction order) within D-to-T events. We then show how this NN can be used to pre-process PG data measured with a CC, for the first time, during the delivery of a clinical proton therapy beam at full clinical dose rates. We showed that the NN pre-processing can help to 1) improve the quality of data (by removing false events) and 2) improve the quantity of good events used for reconstruction (by properly ordering true event interactions and recovering DS events from D-to-T events), both of which help to improve images of PG emission that occurs during clinical proton RT delivery. We believe the previous studies and the NN studies presented in the paper have only scratched the surface of what is possible for PG data and image processing and that the applications of NNs and machine learning in general is a new Frontier in CC imaging that could ultimately expand its capabilities and future applications.

## 2 METHODS

### 2.1 Compton Camera

#### 2.1.1 Compton Camera Design

**FIGURE 1 |:**
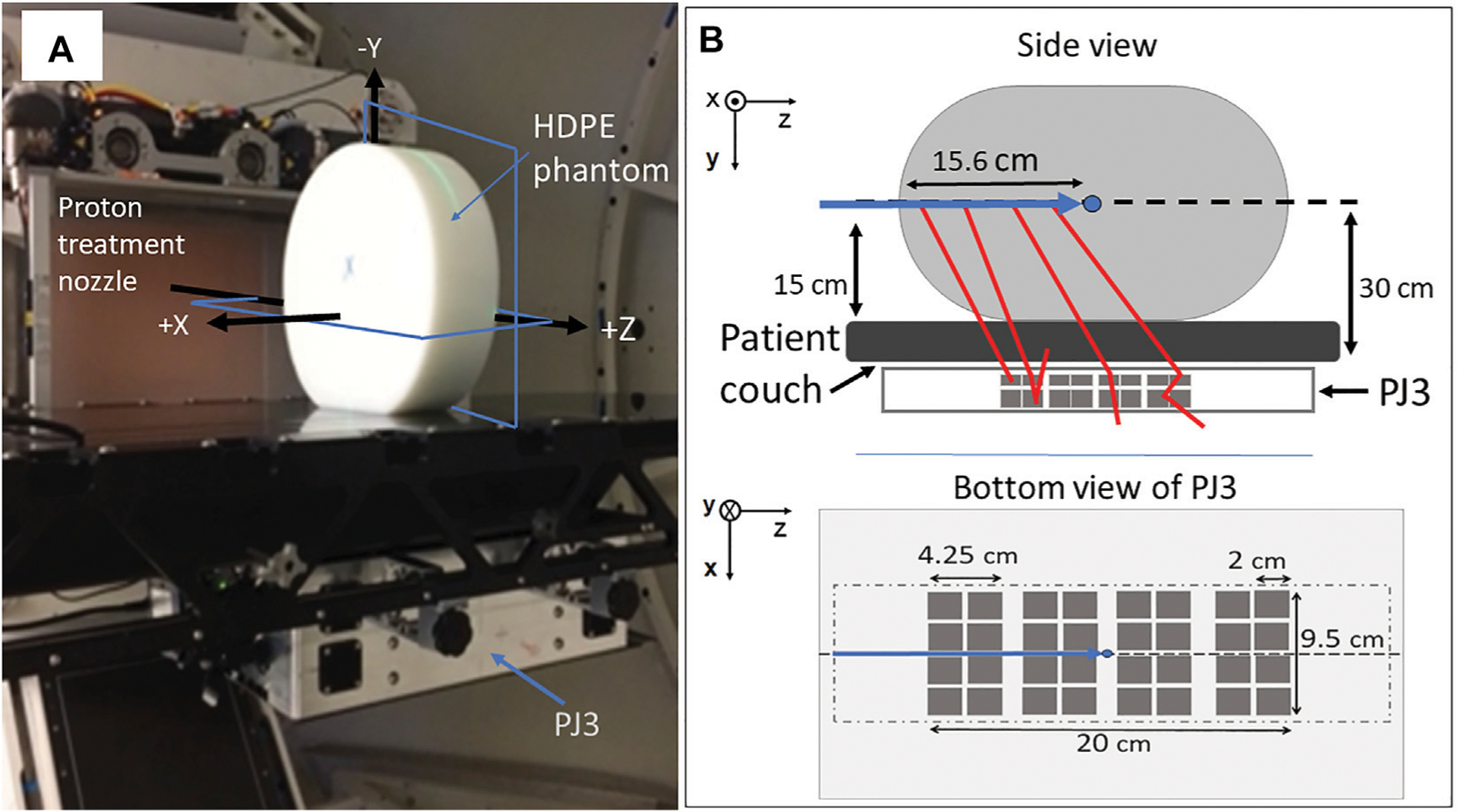
Setup of **(A)** clinical proton pencil beam irradiations for CC measurement of PG emission. **(B)** schematic of the setup showing the positioning of the PJ3 with respect to the beam and including a bottom view of the PJ3 indicating size and positioning of 2 cm × 2 cm CZT crystals (dark gray squares) within the outer box (light gray rectangle) and the outline of the HDPE target (light gray dot-dash line) location above the CZT detectors. Shown in (a) are the locations of the XZ and YZ planes (blue rectangles) of the 2D PG images shown in this paper.

The protoype PJ3 CC (H3D, Inc., Ann Arbor, MI) was used to measure PG emission during clinical proton beam irradiation. As shown in Figure 1, the PJ3 is composed of two detection stages, each containing eight detection modules (16 total) with four cadmium-zinc-telluride (CZT) crystals per module (64 total crystals). Each crystal is attached to a pixelated anode (11 × 11 pixels) that is directly coupled to an application specific intergrated circuit (ASIC) for charge readout. These detectors can provide the positions of interactions with a spatial resolution of about 0.3 mm in 3-dimensions at 662 keV. The CZT crystals have an energy resolution of about 0.4% full width at half max (FWHM) at 662 keV using single pixel events, and about 0.5% FWHM for all events, operated at room temperature [36]. Measured photopeak detection efficiency of the CZT crystals range from 75% at 121 keV, to 1.4% at 2.6 MeV [41].

The crystals in each module are arranged in a 2 × 2 array with a 0.25 cm between the crystals and a 1.0 cm separation between the modules. Each module in stage one (closest to the treatment couch, Figure 1) is composed of 2.0 cm × 1.0 cm × 2.0 cm crystals, and each module in stage two is composed of 2.0 cm × 1.5 cm × 2.0 cm crystals. The distance between the modules in stage one and stage two was 2.5 cm. The detector crystals and the associated electronics of the PJ3 CC are enclosed in a 1.25 mm thick aluminum case along with an electronic interference reduction and heat management system. Further details of the PJ3 design can be found in Maggi et al [34], Panthi et al [32], and Polf et al [35].

#### 2.1.2 Data Acquisition and Readout

Each module of the PJ3 CC operates independently of the other modules, with its own triggering and data-acquisition system. Each module has only one data acquisition (DAQ) and readout channel per module. Therefore, if a PG event is detected in one crystal, the module is triggered and any charge pulse (arising from an interaction) above 50 keV detected on an anode pixel of any of the (four) crystals in the module during a trigger readout cycle will be readout. Due to limitations in the charge detection stability in the ASICs which causes the recorded interaction position to the unreliable for large energy depositions, events that deposit more than 2.7 MeV in a single interaction were excluded from the final data used for imaging and NN processing. The trigger readout cycle for all PJ3 modules consists of a 1.5 μs charge collection window followed by a 4 μs reset time for each pixel that detected an interaction. The data for each interaction that is read out and reported by each module includes: 1) the module and crystal indices, 2) the number of interactions occurring in the module within a trigger readout cycle, 3) the deposited energy of each interaction event, 4) the (*x, y, z*) location of each interaction event, and 5) the timestamp at which each event was read out relative to the beginning of the measurement. A single timestamp is recorded when the module is triggered and this value is recorded as the timestamp for all interactions that occur within the readout cycle. All interactions from a single module with the same timestamp are grouped together in the data file are considered to be a one “event”. For this study, only events recorded within a single module (“intra-module events”) were recorded.

The recorded events were grouped into four types (according to the number of interactions recorded during the triggered readout cycle): 1) single-scatter events (one interaction), 2) DS events (two interactions), 3) TS events (three interactions), and 4) more than three events (four or more interactios). For this study DS and TS events measured during clinical proton beam delivery were used for the PG imaging study. Single-scatter and events with more than three events were removed from the measured data prior to image reconstruction and NN processing.

The individual interactions of any event are recorded in the order that the charge pulse (created by the interaction) is detected by the CZT crystal anode during the readout cycle. This means that an event that occurs closest to the anode in the crystal will most likely be readout first even though it may not be the first (or second) interaction that occurred for that event. This leads to the individual interactions within the event being recorded in the wrong order, which we refer to as a “mis-ordered” (MO) event. A DS event can be readout in two possible interaction orderings leading to one “correctly-ordered” (CO) interaction sequence and one possible MO interaction sequence. For a TS event, with six possible interaction orderings, there is one CO interaction sequence, and five possible MO interaction sequences.

Due to the relatively long length of the PJ3 readout window (1.5 μs), the probability that more than one PG can interact within a detection module during readout increases as PG count rate (due to increasing proton beam dose rate in this study) in the CC increases [35]. An event that contains interactions from more than one PG is referred to as a “false” event in the study. False DS events are composed of two interactions arising from two separate PGs interacting in a detection module within a single readout cycle. Two different types of false TS events can occur in the CC. First three separate PGs may produce single-scatter interactions that are readout as a TS event, and second, a D-to-T event can occur in which a true DS occurs along with a single-scatter interaction from a separate PG and is recorded as a TS event.

### 2.2 Experimental Measurements

For this study, PG data was measured using the prototype PJ3 CC during the delivery of a 150 MeV proton pencil beam to a 15 cm × 30 cm × 35 cm high-density polyethylene (HDPE; C_2_H_4_, *ρ* = 0.97 g/cm^3^) target as shown in Figure 1. The data was measured for dose rates of 20,000 Monitor Units/min (20 kMU/min; 1.22 × 10^9^ protons/s, minimum clinical dose rate at 150 MeV) and 180 kMU/min (1.1 × 10^10^ protons/s; maximum clinical dose rate at 150 MeV), using the Varian Pro-Beam treatment delivery system (Varian Medical Systems, Palo Alto, CA) located at the Maryland Proton Treatment Center (MPTC) in Baltimore, MD. The MU is defined as the clinical unit of dose delivery for radiation therapy machines and is a measure of the number of protons detected by the ionization chambers (determined by its intrinsic charge collected/proton calibration) in the treatment nozzle. For the treatment machine at the MPTC: 1 MU = 3.668 × 10 [6] protons for the 150 MeV treatment beam. For all irradiations, 25 kMU were delivered, equating to 9.17 × 10^10^ protons and delivery times of 75 and 8.33 s at dose rates of 20 kMU/min and 180 kMU/min, respectively. Finally, irradiations (identical setup to the 150 MeV irradiations) were performed and PG data measured with the initial beam energy reduced to 147 and 145.5 MeV to produce a −3 mm and −5 mm shift in the beam range in the HDPE target.

As shown in Figure 1, the PJ3 CC (design details in Polf et al. (2021) [35]) was mounted beneath the patient positioning couch, with the HDPE target placed on the couch directly above the PJ3. The beam was delivered to the center of the HDPE target, located 15 cm above the top of the couch, corresponding to 30 cm from the top of the detector modules in the PJ3. The patient couch was positioned so that the beam path was aligned with the center of the PJ3, and the treatment isocenter was located at a depth of 15.6 cm in the target.

### 2.3 Neural Network Data Processing

#### 2.3.1 Neural Network Desgin

A fully connected NN was constructed with Keras using Tensorflow 2.4.0^43^. A full, detailed description of the construction, training/validation, and testing of the NN was reported by Barajas et al [40]. In brief, the network contains: 1) an input layer which accepts the input data, that consists of a list-mode dataset of all DS and TS events that contains the energy deposited and (x,y,z) coordinates of each interaction of the recorded events, 2) 256 hidden compute layers that use the leaky Rectified Linear Unit activation function [43] and residual skips to perform transformations on the data, and 3) a single output layer which uses the Softmax [43] activation function to return the NN predicted event type classification for each DS and TS event in the input data file.

#### 2.3.2 Neural Network Training and Validation

The NN was trained and validated using PG list mode datasets generated with a Monte Carlo model of the PJ3 and clinical beam delivery, built using the Geant4.10.3 toolkit [44]. The MC generated PG interaction data was then processed by the MCDE [34] model that transforms the MC data according to the response and data acquisition characteristics of the PJ3 CC. The MCDE training datasets included PG emission from 12C (718 keV, 2.0 MeV, and 4.44 MeV), as well as 2.2 MeV H-n capture gammas, and positron emission gammas from several isotopes (11, 10, 9C, 8B, 12N, and 13 N) created in the HDPE phantom during proton irradiation as well as modeling of the Doppler broadening of the PG emission. This produces the final training list-mode dataset containing DS and TS events, as well as, a file that lists whether each event is a True, False, or D-to-T event. Since we did not know what type of gamma interaction was recorded by the CC during PG measurements, our MCDE data used for training and validation included DS events composed of all possible interaction combinations (Compton + photo-absorption, Compton + Compton, and Compton + pair production) and TS events composed of all possible interaction combinations (Compton + Compton + photo-absorption, Compton + Compton + Compton, and Compton + Compton + pair-production) that may occur for consideration by the NN for training. Finally, the interaction order of the DS and TS events are then shuffled such that 50% of the DS events are mis-ordered (and 50% are correctly ordered) and the TS interactions are shuffled so that 16.7% retain the correct interaction ordering, and the remaining 83.3% are shuffled to produce an equal number of the remaining five possible (incorrect) interaction orderings for the TS event. In this way, the final processed list-mode data will provide a PG dataset that accurately models a measured dataset for training the NN to identify the type of each DS and TS event recorded by the PJ3.

For NN training and validation, the MCDE generated: 1) PG interaction list-mode dataset, 2) information on each event type, and 3) information of the correct interaction ordering of each event which are all passed to the NN. The training dataset for this study contained a total of 2.2 × 10^6^ PG events (80% of events for training, 20% of events for validation). Following training and validation, five fully independent (MCDE generated) datasets consisting of 5 × 10^5^ processed PG events (MCDE generated and interaction order shuffled) were used to test the accuracy of the NN. Testing indicated accuracy levels of 87 and 78% for correctly identifying DS (True/False/mis-ordered) and TS (True/False/mis-ordered/D-to-T) event types, respectively.

Following training and validation of the NN, it was used to process PG datasets measured with the PJ3 during the proton beam irradiations described in Section 2.2. Measured DS and TS events (from the CC data files) were input into the trained NN, which then predicted the type and order of the interactions of each event. The NN processing proceeded as follows:

1. Predict if an event is a true DS, false DS, true TS, false TS, or D-to-T event,
2. If the event is a true DS or TS, predict the correct order that the interactions occurred in the CC,
3. If the event is a D-to-T event, predict which two interactions belong to the true DS and predict the correct order in which the DS interactions occurred in the CC, remove the third (seprate PG single-scatter) interaction,
4. If the event is a false DS or TS (three separate PG interactions) remove it from the data.

The events from the measured data file that the NN classified as true DS (including DS events recovered from D-to-T events) and TS events were written to the final “NN Processed” data file with their interactions ordered according to the NN predicted interaction order. An event that was written to the final NN processed data file with the same interaction order as recorded in the raw measured data is referred to as a CO event, while an event in which the NN predicted interaction order is different from that in the raw measured data file is a MO event whose ordering is correctd and therefore referred to as a “Re-ordered” event in the NN processed data.

### 2.4 Image Reconstruction

Image reconstruction of the PG data was performed using the Kernel Weighted Backprojection (KWBP) algorithm, described by Panthi et al [32]. For this study, a full 3D image was reconstructed with KWBP using an 18 cm × 50 cm × 50 cm imaging space. This was processed into 60 separate two-dimensional slices (3 mm thick), with each image slice having 256 × 256 pixels (2 mm pixel size) in the YZ-plane. These PG images were reconstructed using both DS and TS events with a calculated initial energy ranging from either 1) 0.6 MeV–4.5 MeV, 2) 2.0–4.5 MeV, or 3) 4.0–4.5 MeV, where the DS initial energy is taken to be the sum of the energy deposited in the two PG interactions, and the TS initial energy is determined using the gamma ray tracking method described by Schmid et al [23]. All images presented are 2D image slices in the XZ or YZ planes extracted from the 3D dataset. The KWBP reconstructions were performed using an NVIDIA P4000 GPU, with reconstruction times of ~20 s for the number of PG events measured during the delivery of 1 × 10^9^ protons.

Images were reconstructed using the number of events that would be recorded during a clinical treatment delivery of 1 × 10^9^ protons, which we estimated would be the number delivered in the deepest energy layer of a hypo-fractionated treatment field [25, 29]. To do so, the full measured PG datasets for the 150 MeV and −3 mm and −5 mm range shifted beam irradiations were each divided into five independent datasets containing the number of PG events (an event is only included in one data file) that would have been recorded during the delivery of 1 × 10^9^ protons based on the measured PG detection rates at 20 kMU/min and 180 kMU/min dose rates (see Table 1). We then produced images from the raw and NN processed data from the five datasets for each irradiation.

### 2.5 Image Assessment and Range Estimation

A 1D profile along the beam central axis (z = 0 cm), representing the integral of three rows of pixels centered on x = 0 cm, was extracted from the XZ plane images. Additionally, 1D crossfield (lateral) profiles in the *x*-direction in the XZ plane representing the integral of three rows of pixels centered at a depth of z = 10 cm, was extracted for comparison. The PG profiles were compared to depth dose and crossfield profiles extracted from a treatment plan of the 150 MeV pencil beam delivery to the HDPE target (see Supplementary Figure S1) calculated by the MPTC clinical treatment planning system (TPS; Raystation v8A; Raysearch Laboratories, inc., Stockholm Sweden) that was commissioned for clinical proton radiotherapy planning using measured data of the proton beam at the MPTC.

The TPS dose profiles and PG image profiles for raw (prior to NN processing) datasets were normalized to the respective maximum values. The depth of the maximum value (PGmax) and the distal depth (beyond the maximum) at which the profiles fall to 80% (PG80) and 60% (PG60) of the maximum values were determined. The resolution of the profiles is limited to the 2D image pixel size (2 mm), and the PG80 and PG60 values were determined by a linear interpolation between the center position of the voxels before and after the PG profile falls below 80 and 60% of the peak value respectively.

Improvements in our ability to identify the proton beam path in the PG images due to NN processing, were quantified using the image contrast-to-noise ratio (CNR). This is defined as CNR = |S_peak_—S_distal_|/σ_distal_, where S_peak_ is the average image “signal” in the peak intensity region of the individual profiles ranging in depth from 2 cm proximal to 2 cm distal to the PGmax, S_distal_ is the average image “noise” in the individual reconstruction profiles ranging from depths of 21–25 cm that are well beyond the depth of the proton BP. Finally, σ_distal_ is the standard deviation of the image noise values from 21 to 25 cm depth beyond the BP.

1D profiles were extracted from each PG image (using the process described above) and an “average” PG profile (see Supplementary Figure S2) was created as the average PG value (*PG*) of the five individual profiles at each depth (*z*) in the target. Finally, a five-number summary analysis of the PGmax, PG80, and PG60 from each of the five reconstructed images was performed. The median (2^nd^ quartile) value of each metric was determined and the uncertainty in these values was defined as their inter-quartile range (IQR; 3^rd^ quartile—1^st^ quartile).

## 3 RESULTS

### 3.1 PG Measurement and NN Processing

**FIGURE 2 |:**
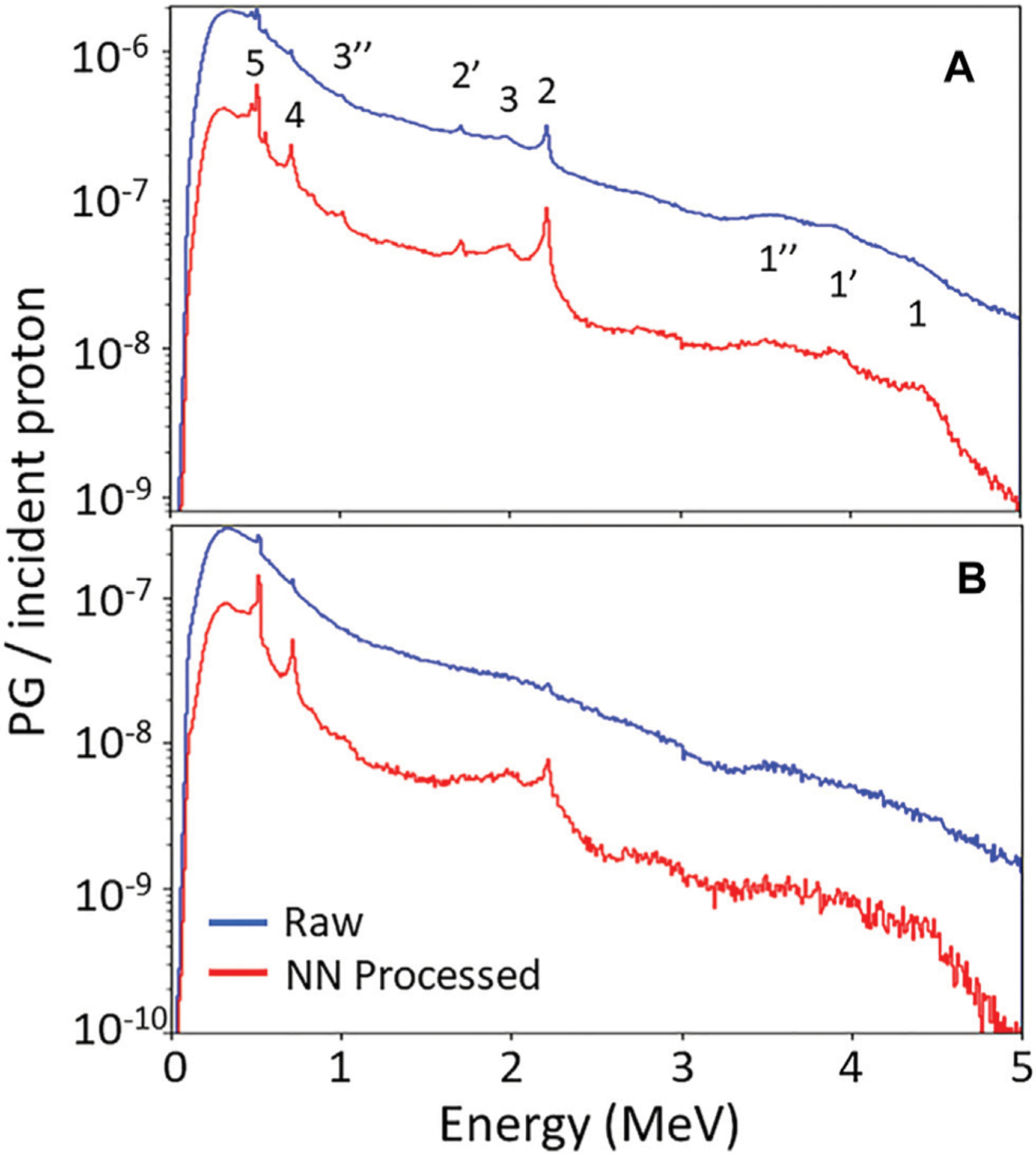
PG energy spectra measured with PJ3 CC during irradiation of the HPDE phantom with a150 MeV proton beam at **(A)** 20 kMU/min and **(B)** 180 kMU/min dose rates for the raw measured data (blue) and following NN processing (red) of the measured data. 1, 1′, 1″ indicate the full absorption (FA), single escape (SE), and double escape (DE) peaks of the 4.44 MeV PG from 12C, respectively. 2 and 2″ indicate FA and SE peak of the 2.22 MeV H-neutron capture gamma, respectively. 3 and 3″ indicate the FA and DE peaks of the 2.0 MeV PG from 12C, respectively. 4 indicates the 718 keV gamma peak from 12C to 5 indicates the 511 keV positron annihilation gamma peak.

Figure 2 shows the energy spectra of PG events (DS + TS) measured by the PJ3 CC during irradiation of the HPDE phantom with the 150 MeV proton pencil beam at 20 kMU/min and 180 kMU/min. PG emission peaks from 12C can be seen at 4.44 MeV, 2.0 MeV, and 718 keV in the raw CC data measured at 20 kMU/min, along with the 2.22 MeV H-neutron capture gamma peak and the 511 keV positron annihilation gamma peak. At 180 kMU/min dose rate, the distinct gamma emission peaks have almost completely disappeared in the raw measured data spectrum with only small peaks distinguishable at 511 keV, 718 keV, and 2.22 MeV. However, after the measured data is processed with the NN, the characteristic PG emission peaks become more prominent due to the removal of the false events and conversion of the D-toT events to true DS events for the NN processed measured data at both the 20 kMU/min and 180 kMU/min dose rates. This results in a reduction in the width of the emission peaks for the NN cleaned data. For example, the 2.22 MeV peak at 20 kMU/min the spectra for the raw measured data has a Full-Width-Half-Maximum (FWHM) of ~40 keV, with the FWHM being reduced to ~18 keV for the NN cleaned data. Such improvements help to move the CC measured data towrd being potentially applicable for PG spectrotrmoetry. This can be further illustrated by looking at the ratio of the full absorption (FA) peak intensity to the single escape (SE) peak intensity [FA/SE], for the 2.2 MeV H-neutron capture gamma measured during the 20 kMU/min irradiation. For the raw measured data [FA/SE]_raw_ = 1.02, while after NN processing of the data [FA/SE]_NN_ = 1.63. Since no SE peak can be seen in the raw measured or NN processd data for the 180 kMU/min irradiation, no such comparison could be made howevern the 2.22 MeV, 718 keV, and 511 keV peaks are all much more prominent.

Table 1 shows a breakdown of the PG events measured per proton incident on the HDPE target by the PJ3 CC during delivery of the 150 MeV clinical proton beam. As the proton beam dose rate increases, the total raw data detection rate (DS + TS events) of the PJ3 decreases from a rate of 1.1 × 10^−4^ events/proton at 20 kMU/min, to 2.57 × 10^−5^ events/proton at 180 kMU/min, a factor of 4.3x. The detection rates include the measurement of all types of DS and TS events, and are in good agreement with previously reported PG detection rates with the PJ3 CC^35^. When this raw measured data is processed by the NN, the detection rate of “usable events” for reconstruction (True DS + True TS + DS events recovered from D-to-T events), as identified by the NN, drops only slightly to 9.09 × 10^−5^ events/proton at the 20 kMU/min dose rate, showing that most data recorded by the CC at the lowest clinical dose rate (and below) are still true events. However, at 20 kMU/min dose rate, only 42% of the raw measured true events are correctly ordered DS and TS events that contribute to the reconstructed image. The remaining mis-ordered true and D-to-T events will only contribute noise to the images reconstructed with the raw measured data. When the dose rate is raised to its maximum clinical value of 180 kMU/min, the detection rate of NN processed usable events drops sharply to 1.47 × 10^−5^ events/proton. Furthermore, only 16.9% of those true events are correctly ordered events showing that not only does the total amount of data recorded drop sharply at higher dose rates, but the quality of the recorded data is also significantly reduced.

### 3.2 PG Image Assessment

**FIGURE 3 |:**
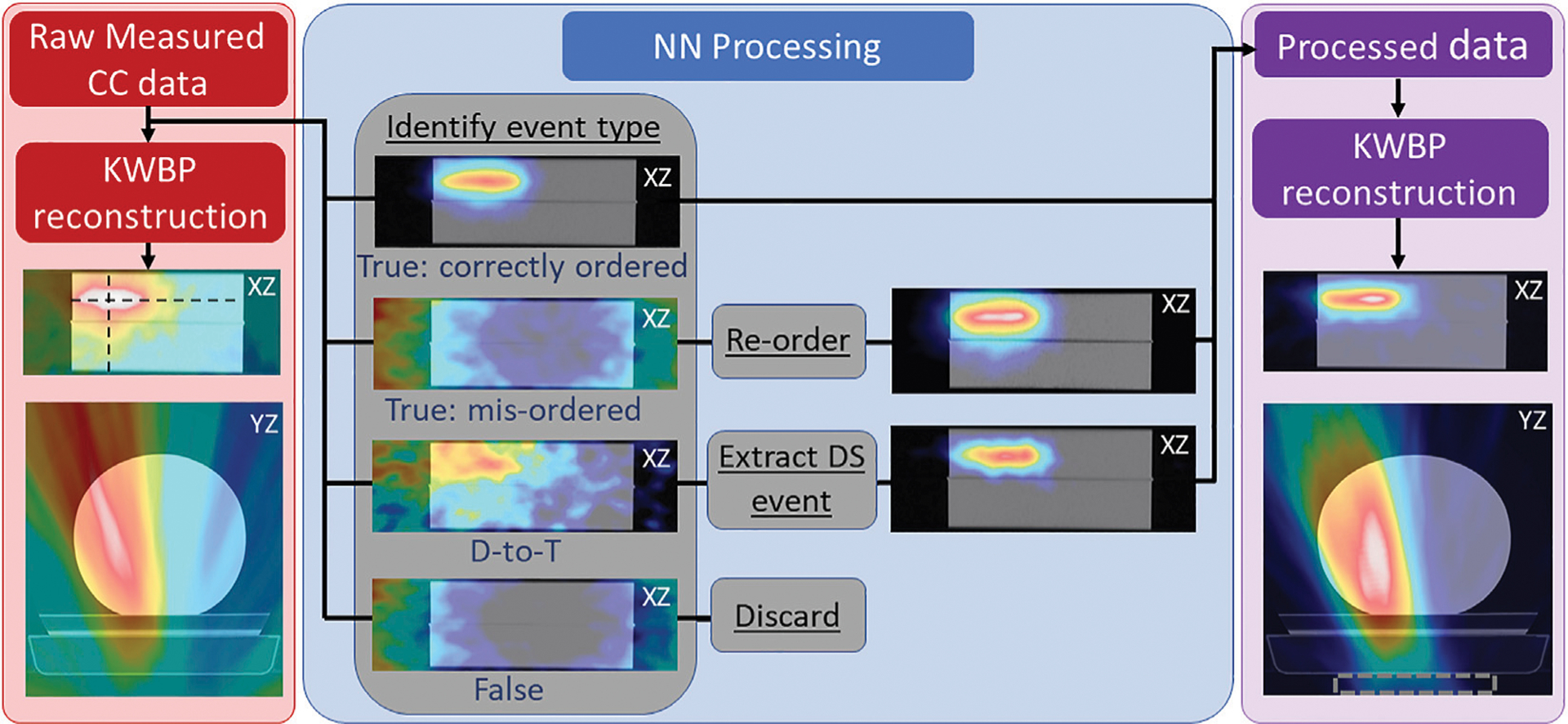
Reconstructed 2D PG image slices in the XZ (coronal) and YZ (axial) planes using the raw measured CC data (measured at 20 kMU/min) overlaid onto a CT scan of the HDPE target (left; red panel), along with a breakdown of the reconstructions of the identified true (correctly ordered and mis-ordered) and false DS and TS events (center; blue panel), and a reconstruction of the DS and TS events after full NN processing (right; purple panel). Dashed rectangle in right panel denotes position of PJ3. Black dashed lines in left panel show location at which 1D profiles (shown in Figure 3) are extracted for beam range analysis. Shown in the center panel are reconstructions of each event type before and after re-ordering the mis-ordered events and before and after extracting (and correctly ordering) the identified DS from D-to-T events to illustrate the effect of NN processing.

Figure 3 shows the PG image reconstructions from raw (DS and TS) events and NN processed events using only the number of PG events that would be measured during the 20 kMU/min proton beam delivery of 1 × 10^9^ protons (according to the detection rates in Table 1). Images were reconstructed using only PGs with initial energies from 0.6—4.5 MeV. Immediately visible is the large stretching artifact in the *y*-direction (perpendicular to the CC) in the YZ plane. This is due to a lack of parallax provided by our single CC in the imaging space [33]. Also, we see a large PG signal in the same location as the proton beam location (see Supplementary Figure S1) in the XZ plane in the raw data image, but a visualization of the end of the beam range is not possible due to the high background noise throughout the image. However, in the XZ planar image reconstructed from the NN processed data, the path of the proton beam and its end of range can be identified and localized as the PG image is localized to the path of the proton beam and the noise level in the image has been drastically reduced.

The center panel of Figure 3 shows the role that mis-ordered true [42], D-to-T, and false events play in the reconstructed raw image data. The correctly ordered true events are the only event type that contribute to the PG emission signal in the image of the raw measured data. The D-to-T events produce a large, diffuse signal on the (left) side of the target that the proton beam irradiates, but no clear image of the beam path is visible [43]. The mis-ordered and false events only produce a “ring” artifact around the edges of the image that is characteristic of these event types in Compton imaging [45]. However, by identifying the D-to-T events and extracting (and correctly ordering) the true DS, and by identifying the correct interaction ordering and re-ordering the mis-ordered events, these two event types now produce a clear image of the beam position and path.

**FIGURE 4 |:**
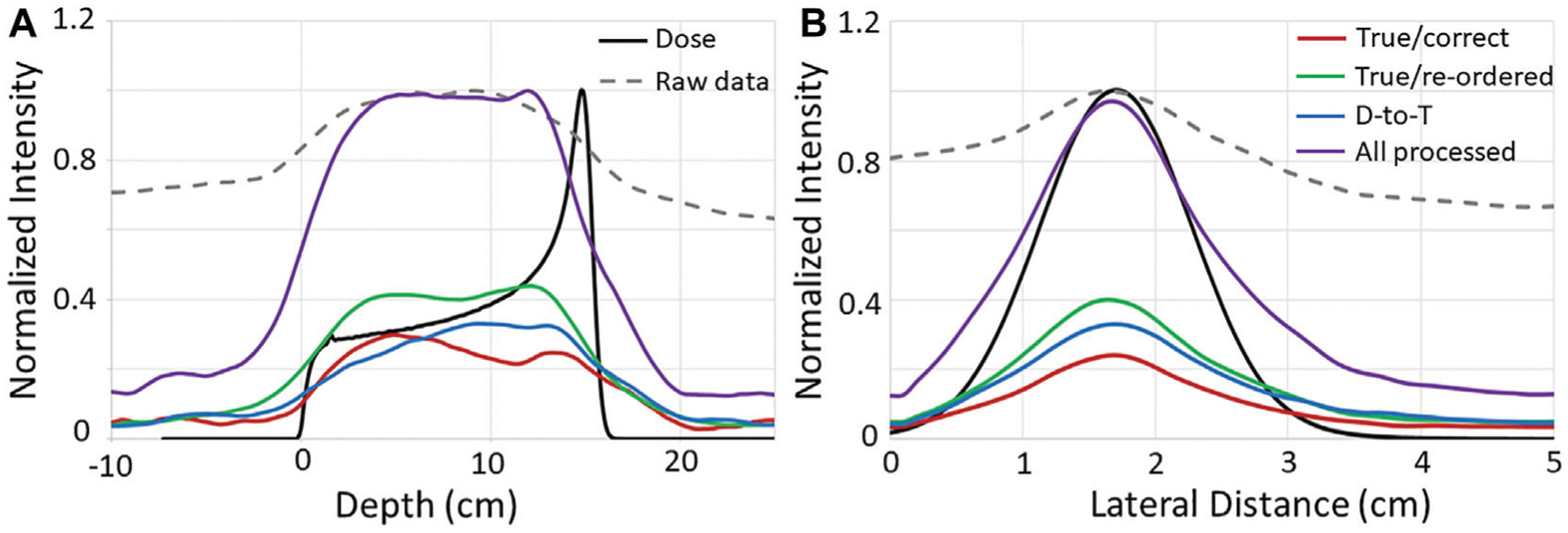
1D **(A)** depth and **(B)** crossfiled profiles from the PG images (shown in Figure 3) reconstructed with the raw and NN processed data, as well as from the proton beam dose profiles. Raw PG data and dose profiles are normalized to their respective maximum values, and NN processed data profiles are normalized to the maximum of the “All processed” profiles. Depth and lateral distance values of zero along the horizontal axis represent the edge of the target.

This shows that these events can be recovered and provide useable data that can improve the final image as shown in the right panel of Figure 3. To further illustrate this, extracted 1D profiles in depth (*z*-direction) and laterally (*x*-direction) are plotted along with depth dose profiles and crossfield (lateral) profiles of the proton beam in Figure 4. These profiles show that the end of the PG emission range is visible in the images reconstructed from each NN processed event type and that the distal edge of the PG signal correlates well with the end of the beam range, and the crossfield profiles of the PG images correlate well to the proton beam crossfield profiles for each NN processed event type.

The improvement to the images reconstructed with the NN processed data can be quantified by the CNR values shown in Table 2. As can be seen, the CNR improves for the NN “All processed” images by a factor of 5.3x and 8.1x over raw data images for the 20 kMU/min and 180 kMU/min data, respectively. In fact, the CNR increases from a factor of 1.7x up to a factor of 7.2x for images reconstructed with each individual type of NN processed data over the raw data images. Conversely, as the range of PG energies used for reconstruction is restricted to include only 4.44 MeV PGs [9, 46] emitted from 12C, the CNR of the images decreases. This CNR drop is due mostly to a significant drop in the number of PGs used for reconstruction. For 0.6–4.5 MeV PGs the total raw and NN processed events are 43,370 and 13,790 for the delivery of 1 × 10^9^ protons at 180 kMU/min. However, as the PG energy range is reduced to 2–4.5 MeV the total raw and NN processed events drops to 5,823 and 2,428, respectively. For PG energies from 4–4.5 MeV, the total raw and NN processed events further decrease to 1,049 and 512, respectively.

**FIGURE 5 |:**
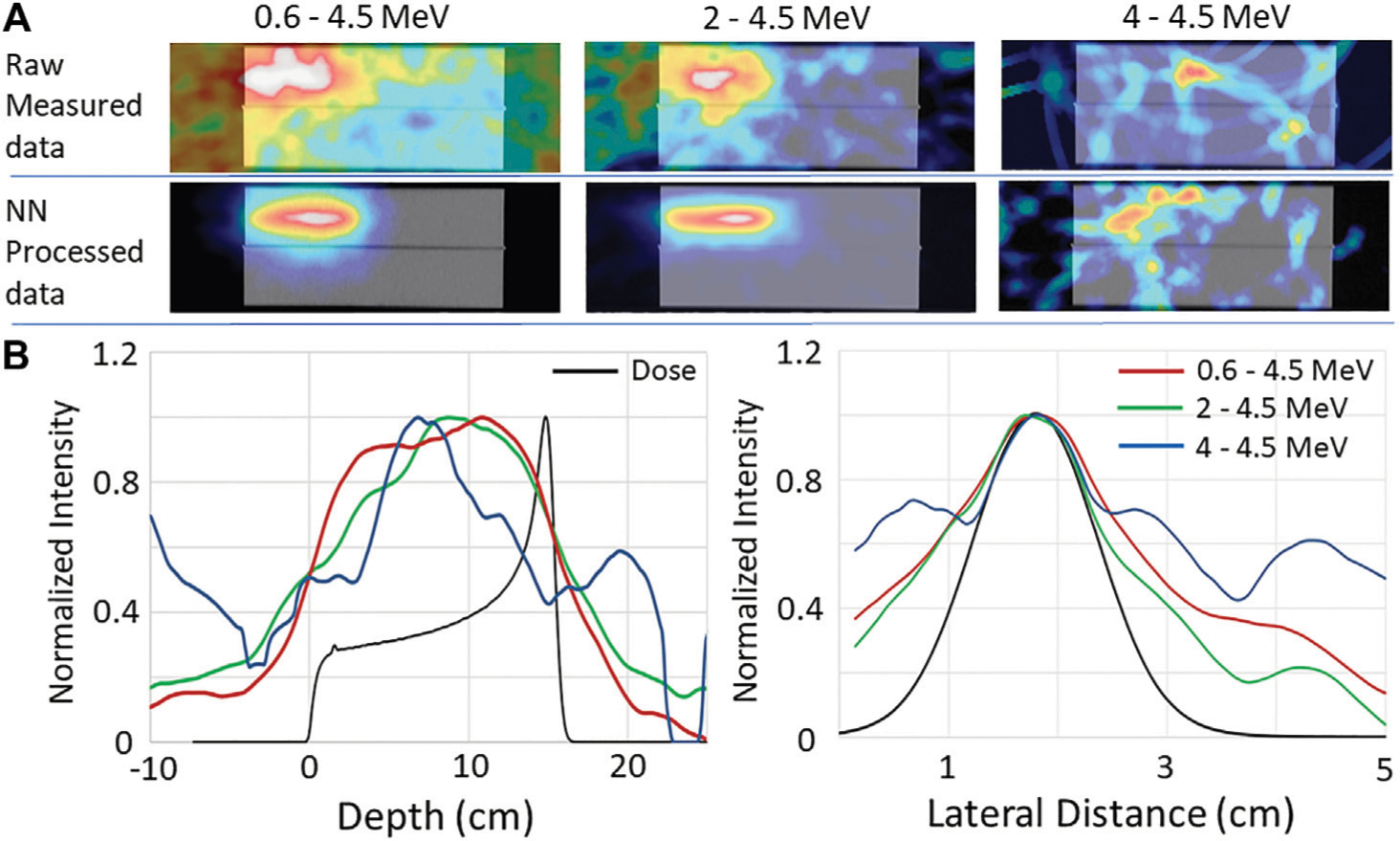
**(A)** Images reconstructed using 180 MeV MU/min raw and NN processed data measured during the delivery of 1 × 10^9^ protons with PG initial energy ranges restricted to 0.6–4.5 MeV, 2–4.5 MeV, and 4–4.5 MeV. **(B)** 1D depth and crossfield profiles (extracted from the same locations as indicated in Figure 3) compared to the depth and crossfiled profiles for the 150 MeV proton pencil beam extracted from TPS calculated treatment plan.

The effect that the drop in PG numbers has on the images of PGs measured at 180 kMU/min is illustrated in Figure 5. As can be seen, the proton beam path is not discernable in the images of the raw measured data for any of the investigated energy levels. However, for the 0.6–4.5 MeV and 2–4.5 MeV energy windows a clear image of the beam path in the target is visible for the NN processed data. However, the lack of events in the energy window from 4–4.5 MeV causes the image of the beam path to disappear for the NN processed data. As can be seen in Figure 5B, the depth dose and lateral 1D profiles extracted from the images reconstructed from the NN processed data, agree well with the dose profiles extracted from the TPS calculation (Supplementary Figure S1) of a 150 MeV pencil beam irradiating the HDPE target for the 0.6–4.5 MeV and 2–4.5 MeV energy windows. However, due to the sharp drop in the number of PG events, the good agreement between the PG and dose profiles is lost in the 4–4.5 MeV energy window.

### 3.3 Range Shift Detection and Uncertainty

**FIGURE 6 |:**
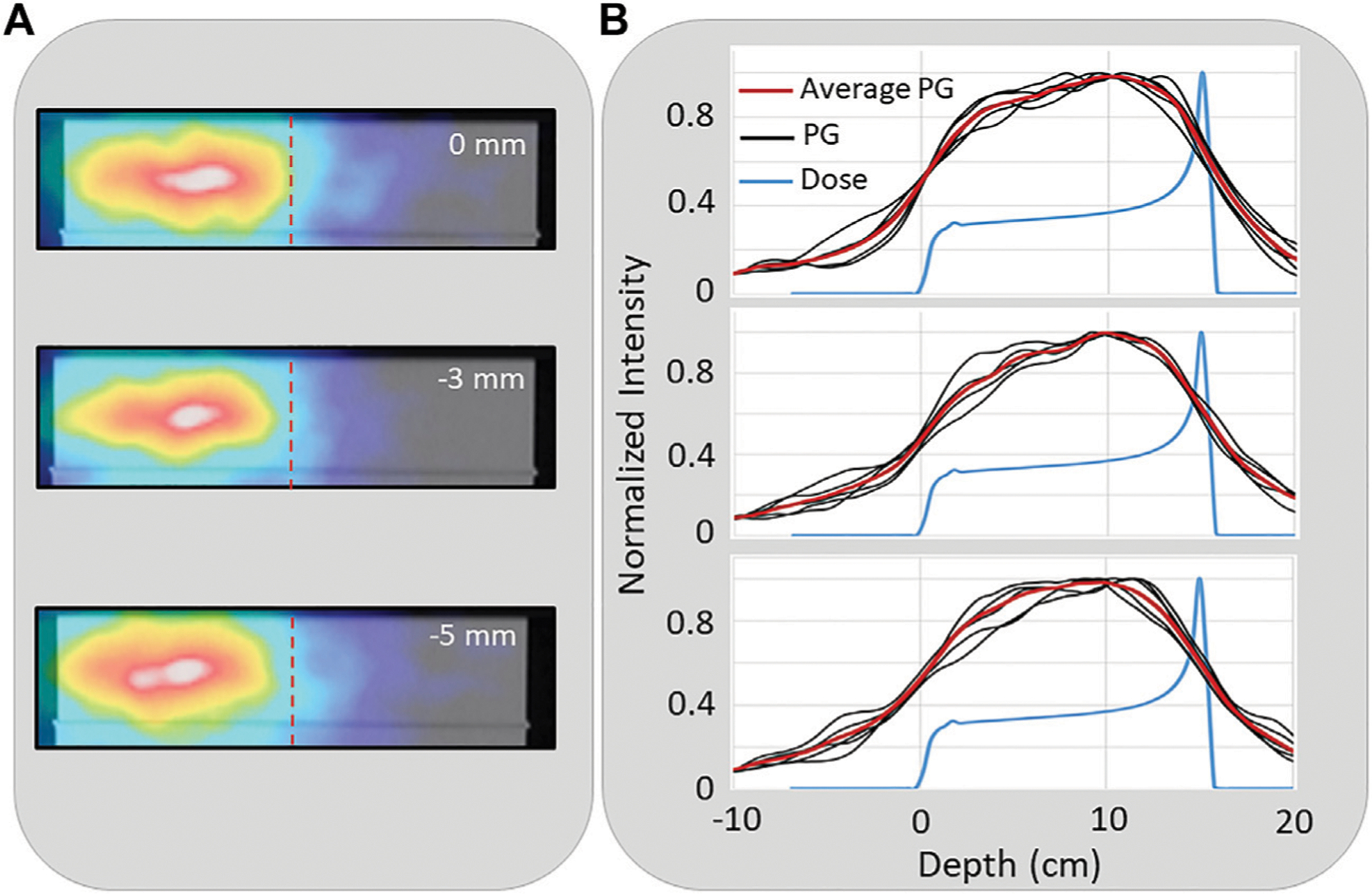
**(A)** An example 2D reconstruction of PG emission measured during the delivery of a 150 MeV proton beam (0 cm) and with the range shifted by −3 mm, and −5 mm. Dashed vertical line indicates depth of distal 80% of the proton depth dose profile in the target **(B)** The 1D profiles extracted from five independent PG images reconstructed from five independent measured PG datasets along with the average PG profile for the full range (top), −3 mm (middle), and −5 mm (bottom) range shifted beams.

Figure 6 shows 2D images in the XZ plane from a single (of the five independent) NN processed PG dataset from the delivery of 1 × 10^9^ protons at 180 kMU/min (0.6–4.5 MeV energy range). For the full range and the −3 mm and −5 mm range shifted beams, a shift in the PG image can be seen in correlation with the proton beam range shift. Also, plotted are the 1D profiles from each of the five images reconstructed (from the five independent datasets) for each beam range along with the average of the five independent profiles. This shows how much variation there is in the 1D profiles extracted from images that are reconstructed from PG emission measured during the delivery of 1 × 10^9^ protons.

**FIGURE 7 |:**
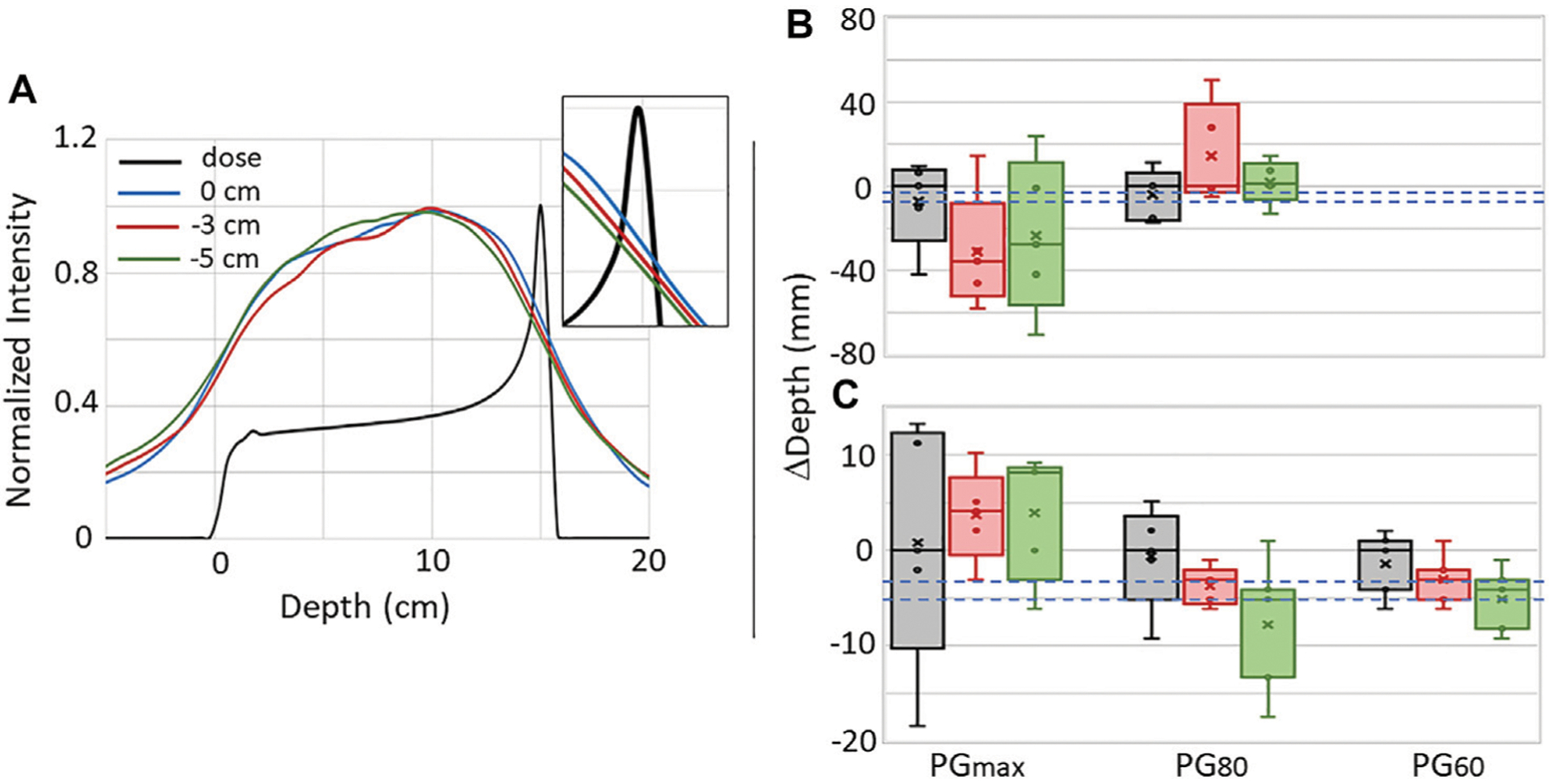
**(A)** The average 1D depth profiles for five independent PG images reconstructed with the NN processed data using 0.6–4.5 MeV PGs measured during the delivery of 1 × 10^9^ protons for the full range (0 cm) and −3 mm and −5 mm range shifted beams. Inset shows close-up view to illustrate the shift in PG profiles at the depth of the distal dose falloff. Box-and-whisker plots for the PGmax, PG80, and PG60 for the five independent images reconstructed with the **(B)** raw and **(C)** NN processed data are shown. In each plot, circles (o) represent individual data points, crosshatches (**×**) represent the mean of the five data points, the line inside the box represents the median (second quartile [Q2]), the box height represents the interquartile range (IQR) extending from the first quartile (Q1) to the third quartile (Q3), while the whiskers represent the minimum and maximum values. Dashed blue lines represent the expected −3 mm and −5 mm shifts.

Figure 7A shows the average 1D PG depth profile for the full range (0 cm), and the −3 mm, and −5 mm shifted beams extracted from images reconstructed from the 180 kMU/min datasets with a PG energy range of 0.6–4.5 MeV. A shift in the average PG profiles can be seen that correlates with the shift in the beam range. To further study whether the PG profiles can be used for proton beam range predictions, five-number summaries of the PGmax, PG60, and PG80 values of 1D depth profiles from each of the five images reconstructed with the raw and NN processed data are shown in Figures 7B,C. Due to the high background in the raw data images (similar to that seen in Figure 4 for the 20 kMU/min data), PG60 values could not be extracted. Even though a shift can be seen in the distal falloff of the average 1D profiles, no correlation can be seen between the beam range shifts and the median and mean shift of the PGmax and PG80 for the raw data. Plus there is a large uncertainty in these values as seen by IQRs ranging from 7.5 mm up to 67.6 mm. While there is still no correlation between the median and mean shifts in the PGmax for the NN processed data, we do see in Figure 7C that the mean and median shifts for PG80 and PG60 do shift in the same direction as the −3 mm and −5 mm range shifts. In fact, for PG60, the median shift values were −2.9 mm and −4.8 mm for the −3 mm and −5 mm shifted beams, respectively. The uncertainty (IQR) in the PG60 shift is 4.8, 3.7, and 4.7 mm for the full range, −3 mm shifted, and −5 mm shifted beam, respectively with a “minimum-to-maximum” value spread (as seen by the whiskers in Figure 7C) of up to 7.5 mm.

## 4 DISCUSSION

The data presented show how NN processing of measured CC data can improve the reconstructed PG images, which agrees with previously published studies [26, 38, 39]. In a previous study [40], we have shown that the NN used in this study can not only detect true and false events, but can also simultaneously predict interaction order of the true events with an overall accuracy of 84%. As shown in Figure 3, this type of processing can be used to remove the false DS/TS events and to recover PG events for use in image formation that would otherwise only have contributed noise to the image. This leads to a large reduction in image PG background, which improves the correlation of the PG image to the delivered dose ditributions as seen in Figure 4. Additionally, we show for the first time (to our knowledge) that the improvements in the PG images made possible with NN processing of the data can also be achieved for PG data measured during the delivery of clinical proton beams at full clinical dose rates. As seen in Figure 5, the PG image produced from CC data measured at the highest clinical dose rate does not produce a clinically usable image that can be used to identify the beam path and end of range in the phantom (patient). However, after this measured CC data is processed with our NN, the beam path and end of range can be easily identified in the image.

At the lowest clinical dose rate, a noisy image was reconstructed from the raw data acquired with the PJ3 CC, but as the dose rate was increased to its highest level, the PG image is completely lost in the noise within the raw data in agreement with our previous studies [35]. In fact at the highest clinical dose rate, only ~17% of the raw data are “usable” (correctly ordered true DS/TS) events that contribute to the PG image with the remainder only producing noise that overwhelms the image of the PG emission. However, after NN processing and recovery of mis-ordered and D-to-T events, >55% of the data will contribute to the PG image, with the remaining false events being removed. This increase in usable events and removal of NN identified false events work together to make it possible to reconstruct an image of the path, end of range, and lateral width of the proton beam in the target even at the highest clinical dose rate. The improvement in the image quality was best quantified by the factor of >8x increase in CNR for the images reconstructed by NN processed data compared to the raw data images.

NN processing of the PG data must be balanced against the degradation of the images caused by the loss of PG events used for reconstruction. This can be seen in Figure 5 with the loss of the well defined image as the number of events used for reconstruction is reduced by more than a factor of 40x and 25x for the raw measured data and NN processed measured data, respectively as the initial energy range of the PGs used for reconstruction is restricted from the full energy range (0.6–4.5 MeV) down to only the 4.44 MeV PGs from 12C. This sharp reduction is due to the reduction of intrinsic efficiency of the CC as PG energy increases, as well as the limitations in the current readout electronics which limits the upper energy deposition of any single event to below 2.7 MeV. It is well known that using only PGs with measured initial energies within ranges that correspond to known PG emission lines will improve the correlation of the PG images to the delivered proton beam range [31, 47, 48]. However, tightly restricting the PG energies used for reconstruction can lead to addiational complications, such as the dependence of the PG emission cross section on the proton energy and uncertainties in the recorded PG energy deposited due to detector thermal instabilities or high detector dead time. These difficutlies work to further increase the need to measure more data during any given measurement, especially since current methods of PG image reconstruction such as iterative maximum likelihood or origin ensemble methods and even simple, filtered or kernel weighted back-projection methods are very sensitive to PG statistics [8, 31, 49, 50] and thus the first concern for CC imaging is to detect an adequate number of events. For this study we reconstructed images from PG emission measured during the delivery of the upper limits of the number of protons (1 × 10^9^) that would be delivered for high dose, hypo-fractionated clinical treatments. A single pencil beam delivered for a standard proton treatment would only deliver between ~10^7^–10^8^ protons meaning the number of PGs detected could be up to 100x lower, thus making the reconstruction of images more difficult and further stressing the need for high PG detection efficiency and event recovery with NN processing of the data.

With the improvement to the number of PG events and data quality that was made possible by our NN processing, beam range shifts as small as 3 mm could, on average, be seen in depth profiles extracted from images reconstructed with PG data measured during the delivery of a single high dose clinical pencil beam. However, the ability to predict range shifts from 1D profiles extracted from images reconstructed with the NN processed data was still less precise than that demonstrated with 1D imaging methods such a slit-camera [10, 18]. From analysis of the uncertainty in the extracted depth profiles, at the highest clinical dose rates the smallest shift that could be detected from any single measurement was ~5 mm based on the shift of PG60. In fact, based on the spread in the PG60 (NN processed data) values, as shown by the whiskers in Figure 7C, we would say that the smallest shift that can be determined with adequate confidence for clinical evaluation with the current PJ3 prototype would more likely be ~7.5 mm. The uncertainty in the PG based range determination is again driven by the low efficiency for detecting usable DS and TS events, even after NN processing of the data. This uncertainty could potentially be reduced by employing noise reduction techniques similar to those used with 1D slit cameras such as, aggregating the PG signal from several spots, comparing measured results to high statitics Monte Carlo simulations, or Guassian smoothing of the extracted 1D profiles [10, 18]. Additionally, the low number of detected usable PG events combined with the lack of parallax provided by a single camera act together to limit CC based PG imaging to a 2D imaging technique.

To truely make online proton (and heavier ion) beam imaging and verification possible, it is necessary to improve the final images we are able to construct. This will need to be done in two primary ways: 1) by increasing the quantity of the measured particles/signals druing treatment delivery, and 2) improving the quality of data used for image reconstruction. Boosting the measured signal can be accomplished by further improving the physical detectors used for acquisition [35, 51], as well as potentially expanding the types of secondary particles (beyond gammas) to include others produced during proton and ion beam therapy, such as through secondary particle tracking [52–54] or interaction vertex imaging [51, 55, 56]. Data quality imrovements as well as improvements to the final reconstructed images will be driven by the advancements in machine learning and other forms of artificial intelligence.

We believe the results presented in the work demonstrate the potential of machine learning and NN based processing of CC data to improve PG imaging for the purpose of proton beam range verification. Thus, we conclude that further development into improved detection systems for CCs and further application of NNs and machine learning will help to move CC imaging for PG range verification closer to clinical application.

## Supplementary Material

SupplementalDocument

## Figures and Tables

**TABLE 1 |:** Detected PG events per proton for raw and NN processed measured data.

Dose Rate (kMU/min)	Raw data (×10^−6^)		NN processed data (×10^−6^)						
	DS	TS	DS			TS			
	Total	Total	True	True	False	True	True	D-to-T	False
			Correct order	Mis-ordered		Correct order	Mis-ordered		
20	90.04	20.31	35.39	35.62	19.03	2.35	11.71	5.8	0.45
180	17.76	7.92	4.01	4.03	9.72	0.33	1.74	4.58	1.27

**TABLE 2 |:** Contrast-to-Noise (CNR) values for images reconstructed with raw and NN, processed data.

Dose Rate (kMU/min)	Energy range (MeV)	Raw Data	NN processed data			
		All	True	True	D-to-T	All
			Correct order	Re-ordered		
20	0.6–4.5	56.3	160.3	99.6	281.6	300.5
180	0.6–4.5	30.1	65.5	142.1	215.5	245.4
180	2–4.5	11.6	–	–	–	219.2
180	4–4.5	0.5	–	–	–	1.8

## Data Availability

The raw data supporting the conclusion of this article will be made available by the authors, without undue reservation.
