# Supplementary material for: Applications of Machine Learning to Improve the Clinical Viability of Compton Camera Based in vivo Range Verification in Proton Radiotherapy
Source: Front Phys. Author manuscript; Available in PMC 2022 Sep 16. (PMC9481064; doi:10.3389/fphy.2022.838273)
Supplement: SupplementalDocument [file NIHMS1835473-supplement-SupplementalDocument.pdf]

Supplemental Materials for:

Applications of machine learning to improve the clinical viability of Compton camera based in vivo range verification in proton radiotherapy.

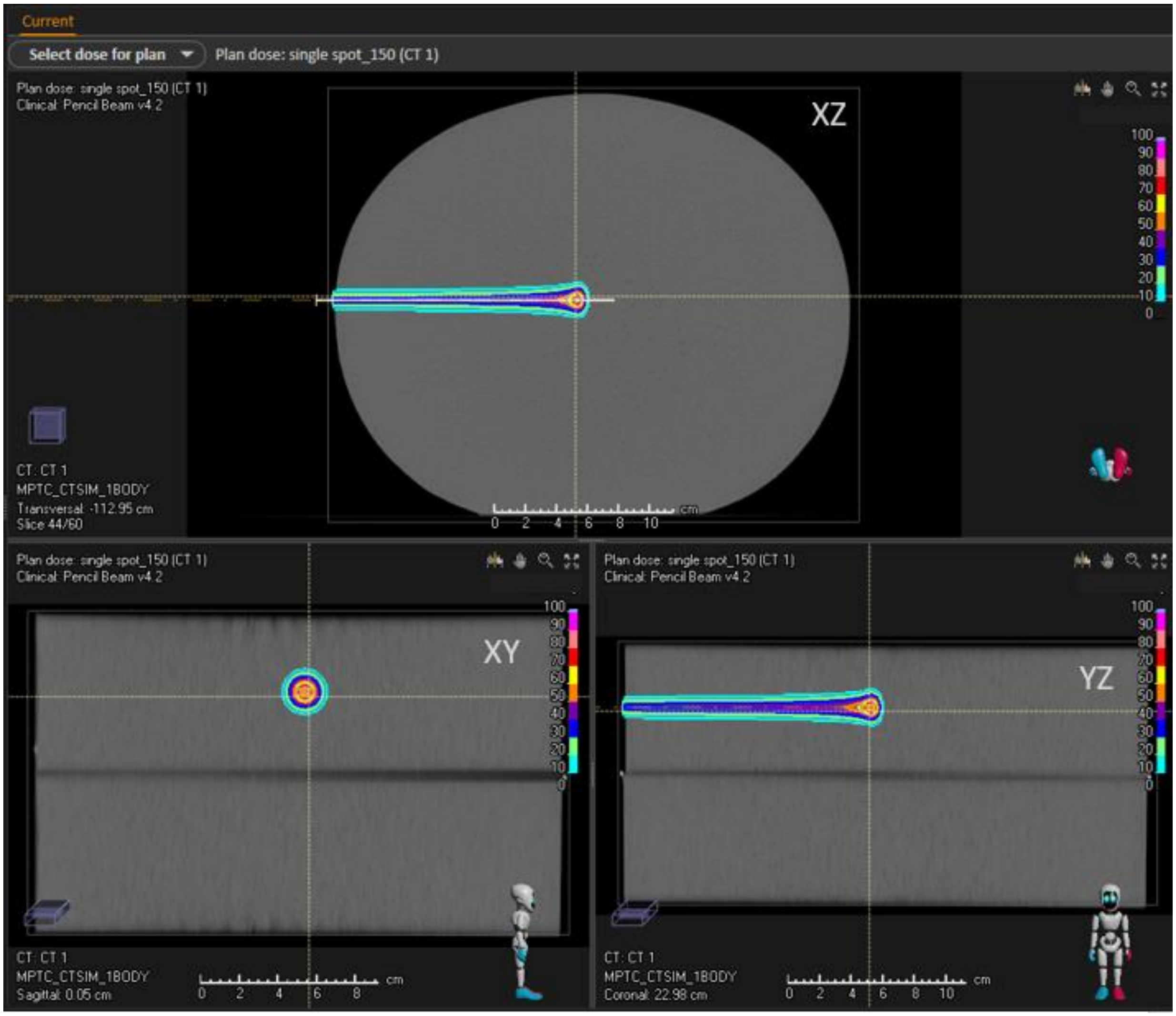

Supplemental Figure S.1: Planned 150 MeV proton pencil beam dose delivery to the HDPE phantom using the Raystation Treatment Planning system (Raystation v8A; Raysearch Laboratories, inc., Stockholm Sweden) commissioned for patient treatment planning at the MPTC. Treatment plans with a single pencil beam were also created for the 147 MeV (-3 mm range shift) and 145.5 MeV (- 5mm range shift). The plans for each beam energy were created to deliver 25,000 MUs.

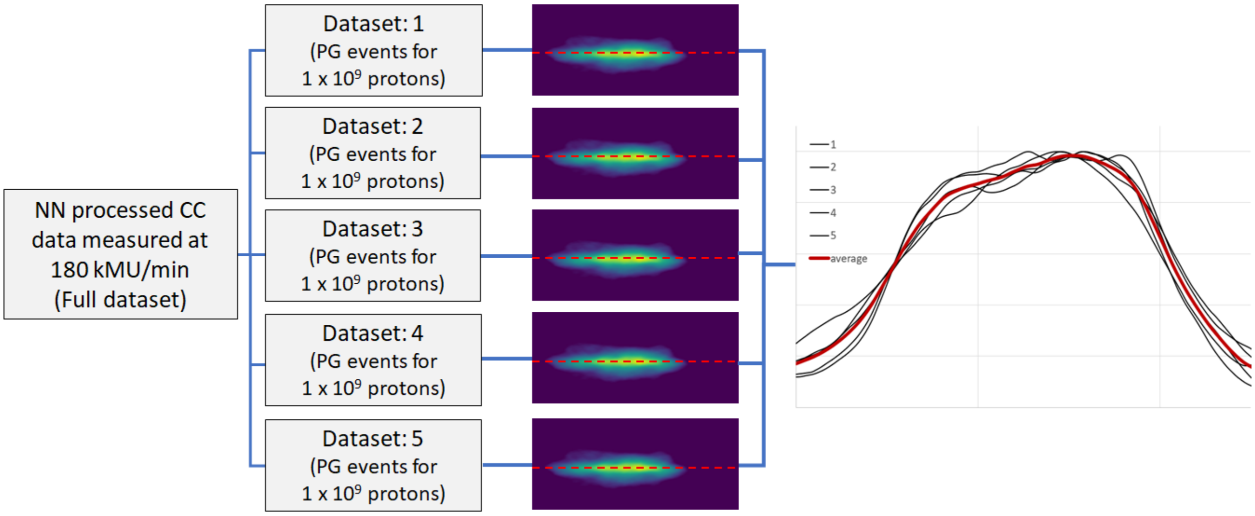

Supplemental Figure S.2: A schematic of the process used to calculate the "average" PG profile. Five independent datasets were created from the NN processed dataset measured at 180 kMU/min. Then five independent PG images were created using the number of PG events recorded for 1 x 10^9 protons delivered. 1D PG profiles were extracted from each image (black lines) and the "average" of the five profiles created (red line). The "average" PG profile (red line) was created as the average PG value ($\overline{PG}$) of the five individual profiles as a function of depth (x) is calculated as: $\overline{PG}(x) = \frac{1}{5}\sum_{k=1}^{5} PG(x)$.
